# Early warning strategies for corporate operational risk: A study by an improved random forest algorithm using FCM clustering

**DOI:** 10.1371/journal.pone.0318491

**Published:** 2025-03-11

**Authors:** Xini Fang

**Affiliations:** School of Safety Science and Emergency Management, Wuhan University of Technology, Wuhan, Hubei, China; Xinyu University, CHINA

## Abstract

To enhance the accuracy and response speed of the risk early warning system, this study develops a novel early warning system that combines the Fuzzy C-Means (FCM) clustering algorithm and the Random Forest (RF) model. Firstly, based on operational risk theory, market risk, research and development risk, financial risk, and human resource risk are selected as the primary indicators for enterprise risk assessment. Secondly, the Criteria Importance Through Intercriteria Correlation (CRITIC) weight method is employed to determine the importance of these risk indicators, thereby enhancing the model’s prediction ability and stability. Following this, the FCM clustering algorithm is utilized for pre-processing sample data to improve the efficiency and accuracy of data classification. Finally, an improved RF model is constructed by optimizing the parameters of the RF algorithm. The data selected is mainly from RESSET/DB, covering the issuance, trading, and rating data of fixed-income products such as bonds, government bonds, and corporate bonds, and provides basic information, net value, position, and performance data of funds. The experimental results show that the model achieves an F1 score of 87.26%, an accuracy of 87.95%, an Area under the Curve (AUC) of 91.20%, a precision of 89.29%, and a recall of 87.48%. They are respectively 6.45%, 4.45%, 5.09%, 4.81%, and 3.83% higher than the traditional RF model. In this study, an improved RF model based on FCM clustering is successfully constructed, and the accuracy of risk early warning models and their ability to handle complex data are significantly improved.

## 1. Introduction

Operational risk refers to the various uncertainties and potential losses an enterprise may encounter during its operations. Such risks may stem from market fluctuations, financial issues, research and development (R&D) failures, or suboptimal human resource management, among other factors [1]. The presence of these risks not only jeopardizes the survival and development of the enterprise but can also have far-reaching impacts on shareholders, employees, customers, and even the entire economic system. Although risk management is an indispensable part of corporate operations, existing early warning systems often exhibit limitations [2,3]. For instance, these systems may rely on a single risk assessment model, lacking the flexibility to adapt to dynamic market environments, or excessively depend on historical data, neglecting the identification and assessment of emerging risk factors. Moreover, current systems may fall short in response speed and warning accuracy, potentially causing enterprises to miss opportunities for risk prevention or mitigation. The existing corporate operational risk early warning system usually relies on a single risk assessment model, such as Logistic regression and support vector machine (SVM) [4]. Traditional data preparation methods usually include missing value filling, outlier processing, and standardized processing, but these methods may not be flexible enough to adapt to different data distribution characteristics when dealing with complex data [5].

Many scholars have employed various statistical methods and machine learning algorithms in previous studies to construct corporate risk early warning models [6]. Certainty and uncertainty are ubiquitous and permeate almost every aspect of daily life. Moreover, it is challenging to accurately understand and deal with uncertain and ambiguous information in practical decision-making problems [7,8]. For instance, logistic regression, SVM, and traditional random forest (RF) algorithms have been widely applied in this field. These methods have improved the accuracy and efficiency of risk warnings to a certain extent. However, most studies employ subjective weighting or simple statistical methods to determine the weights of risk assessment indicators, ignoring the interrelationships and relative importance of these indicators. This oversight leads to insufficient sensitivity of the models to key risk factors [9,10]. Traditional data preprocessing methods fail to fully explore the intrinsic structural characteristics of complex and highly heterogeneous data, leading to reduced model prediction performance.

Given the complexity and variability of operational risks and the shortcomings of existing early warning systems, this study seeks to develop a more accurate and responsive risk early warning system. The motivation for this study arises from the necessity to enhance corporate risk management capabilities and address the deficiencies of current early warning systems. This study aims to improve the prediction accuracy and response speed of the warning system by integrating advanced data analysis techniques and risk assessment methods. This improvement holds significant academic value by exploring new applications of data analysis methods in risk management. Moreover, it has substantial practical significance by helping enterprises identify and manage risks more effectively, thus enhancing their competitiveness and market adaptability.

Unlike hard clustering algorithms such as K-means and DBSCAN, Fuzzy C-Means (FCM) allows data points to belong to multiple clusters, each with a membership degree. This soft clustering capability enables FCM to better handle overlaps and uncertainties between data points, thus enhancing the accuracy and robustness of classification. FCM assigns data points through membership values, effectively handling noise and outliers in the dataset, whereas hard clustering algorithms like K-means are more susceptible to interference when dealing with such data. In comparison, the parameter selection for algorithms such as K-means and DBSCAN is relatively fixed, leading to poorer adaptability.

As global market competition intensifies and the corporate operational environment becomes increasingly complex, the risks and challenges faced by enterprises also multiply. Existing early warning systems may rely too heavily on single risk assessment models or historical data, lacking flexibility and adaptability, which results in insufficient accuracy and response speed in early warnings. Addressing these issues, this study aims to enhance the accuracy and response speed of the early warning system by developing a novel system that combines the FCM clustering algorithm with the RF model. This study’s main content includes the following. Firstly, based on operational risk theory, market risk, R&D risk, financial risk, and human resource risk are identified as the primary indicators for evaluating enterprise risk. Secondly, the Criteria Importance Through Intercriteria Correlation (CRITIC) weighting method is employed to calculate the weights of these risk indicators, clarifying the relative importance of different risk factors in the overall risk assessment. Subsequently, the FCM clustering algorithm is utilized to preprocess sample data from the Corporate Bankruptcy Prediction database to improve the efficiency and accuracy of data classification. Additionally, the Grid Search method tunes the RF algorithm’s parameters, thus achieving optimal model prediction performance. Finally, this study proposes strategies for preventing and controlling operational risks, providing practical guidance for enterprise risk management. Therefore, an advanced and accurate early warning model for corporate operational risk is constructed by comprehensively applying these methods. The main contribution of this study lies in the development of an improved RF model that integrates the FCM clustering algorithm to enhance the accuracy and response speed of the early warning system. The CRITIC weight method is used to determine the importance of risk indicators, enhancing the model’s prediction ability and stability.

## 2. Related work

In the corporate operational risk early warning field, numerous scholars have conducted in-depth research and proposed various theoretical frameworks and early warning models. For example, Cao et al. (2022) proposed a financial risk early warning method by deep learning algorithms [11]. The research aligned with the goal of this study to improve the accuracy and response speed of risk early warning systems using an FCM clustering-enhanced RF algorithm. Wang et al. (2023) utilized blockchain technology to construct a network public opinion risk management system based on smart contracts [12], tracing public opinion through smart ledgers and risk association trees. Kristanti et al. (2021) identified key factors leading to financial distress in insurance enterprises, such as changes in surplus, premium growth, and enterprise size [13]. These factors were closely related to the financial risks discussed in this study and provided references for selecting and weighing risk assessment indicators. Zhu et al. (2021) proposed a financial risk assessment method based on the Z-Score model [14]. Li et al. (2023) introduced an optimized Back Propagation (BP) neural network model as a financial early warning model, emphasizing its high predictive accuracy [15]. Song et al. (2023) used the K-means clustering algorithm to classify the financial status of enterprises. They performed factor analysis to obtain eight common factors for constructing the early warning model: debt-paying ability, profitability, operational capability, growth ability, cash flow, value creation, creativity, and equity structure [16]. Lee (2023) focused on financial indicators such as cost and expenses, debt-paying ability, and operational capability to evaluate the operational status of information service enterprises, providing a basis for management decisions. Lee’s research indicated that the gross profit margin of information service enterprises was influenced by several factors, encompassing operational management, R&D capabilities, debt-paying ability, and enterprise size [17]. Chen (2024) employed an improved Kaufman-Merton-Voss (KMV) model for quantitative early warning analysis of bond default risk [18], which was consistent with the approach of this study using an improved RF model for corporate operational risk early warning. Both aimed to enhance the accuracy and response speed of risk prediction by using and optimizing advanced models, providing more reliable risk management tools for enterprises. Weng et al. (2024) proposed establishing an internal control system for corporate financial risks from multiple perspectives. It demonstrated that a comprehensive internal financial risk control system [19] could classify and assess the financial risk levels of enterprises based on market, credit, and liquidity risks.

With the advancement of artificial intelligence technology, an increasing number of sophisticated computational models have been applied to the corporate operational risk early warning field. For instance, AIP DeepEnC-GA [20] optimized deep learning models through genetic algorithms to enhance prediction accuracy; StackedEnC AOP [21] utilized stacked encoders to capture deep features of data, strengthening the model’s generalization abilities; DeepAVPTPPred [22] focused on the prediction of time series data, effectively addressing market dynamics; iAFPs Mv BiTCN [23] combined multi-view and bidirectional temporal convolutional networks, improving the processing of complex data; Deepstacked AVP [24] achieved precise prediction of risk factors through deep stacked networks.

The traditional RF algorithm has been widely studied for its application in risk prediction. Sipper et al. (2021) proposed the fundamental theory of the RF algorithm and demonstrated its powerful classification and regression capabilities in various fields [25]. However, Shah et al. (2020) argued that while the RF algorithm excelled in handling high-dimensional data, its reliance on randomness to generate tree models might make it sensitive to data outliers and noise. This sensitivity could potentially affect the stability and generalization ability of the model [26]. To address this issue, this study proposes an improved RF model based on the FCM algorithm. By incorporating fuzzy clustering techniques, the model effectively handles the fuzziness and uncertainty of the data, thereby enhancing the prediction accuracy and stability.

Although significant progress has been made in the field of enterprise operational risk early warning, some limitations and challenges remain. Many existing models rely heavily on historical data and lack dynamism, making it difficult to respond in real-time to the continuously changing market environment. Moreover, these models may underperform when handling complex and high-dimensional data, especially when the data contains noise or uncertainty [27,28]. To overcome these shortcomings, this study proposes an innovative corporate risk early warning system that integrates the FCM clustering algorithm with the RF model. This system can perform comprehensive risk assessments across multiple dimensions and industries, with improved real-time responsiveness and better adaptability to data. Despite the significant progress made by existing studies in the corporate operational risk early warning field, there are still many limitations and challenges. Current research tends to focus on a single dimension or specific industries, lacking comprehensive risk assessment across multiple dimensions and industries. Some models perform poorly in handling complex data and real-time responses. To overcome these shortcomings, this study proposes an innovative early warning system that combines the FCM clustering algorithm with the RF model, achieving multidimensional, cross-industry integrated risk assessment. Specifically, the FCM clustering algorithm can effectively process complex data and identify potential risk groups. The RF model boasts high prediction accuracy and strong interpretability, providing robust support for risk early warnings. Additionally, the system has a high real-time response capability, reflecting market changes and enterprise operations promptly, offering enterprises a more reliable risk management tool.

## 3. Research methodology

### 3.1. Corporate operational risk theory

Corporate operational risk theory is a critical component of strategic planning and decision-making within enterprises. It addresses various uncertainties and potential losses that enterprises may face during their operations. These risks can originate from market fluctuations, R&D failures, financial issues, or deficiencies in human resource management. Categorizing operational risks helps enterprises to identify and address these uncertainties more specifically. Typically, these risks can be classified into market risk, R&D risk, financial risk, and human resource risk.

**Fig 1:**
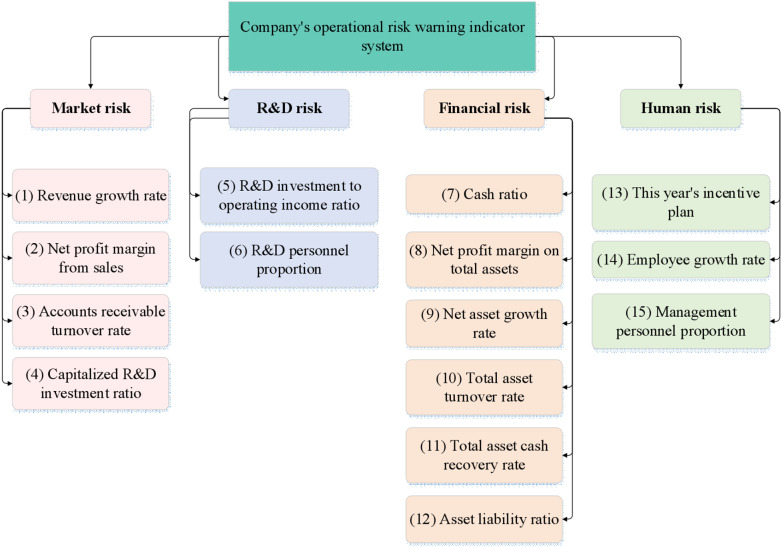
Corporate operational risk early warning indicator system.

Market risk pertains to uncertainties arising from market demand, price fluctuations, increased competition, and other factors. Enterprises must remain sensitive to market trends to promptly adjust their products or services to meet market demands. R&D risk involves uncertainties in exploring new products or technological innovations, including technical feasibility, cost overruns, and innovation failures. Financial risk encompasses issues related to capital acquisition, investment decisions, cash flow management, and credit risk, directly influencing an enterprise’s financial health and ability to sustain operations. Human resource risk focuses on employee recruitment, training, retention, and labor cost management. The abilities and loyalty of employees are crucial for the long-term success of an enterprise. Based on this, the corporate operational risk early warning indicator system is demonstrated in Fig 1.

### 3.2. Data sources and description

The data utilized in this study is primarily sourced from the RESSET/DB database, which encompasses the issuance, trading, and rating data of fixed-income products, including bonds, government bonds, and corporate bonds. Furthermore, RESSET/DB provides fundamental information, net value, positions, and performance data of funds, which are of significant importance for assessing corporate operational risks. Uniform Resource Locator of the dataset: https://resset.cn/index/home/. The dataset comprises operational data from 1,000 enterprises, spanning the period from 2010 to 2020. Based on operational risk theory, this study selects market risk, R&D risk, financial risk, and human resource risk as the main assessment indicators. Specific variables include the revenue growth rate, cash ratio, debt-to-equity ratio, and R&D expenditure, among others. The database is maintained by a professional data collection organization, updated regularly, and goes through a strict quality control process to ensure the accuracy and integrity of the data. It not only covers the historical bankruptcy cases of many enterprises but also contains detailed financial indicators, market information, R&D expenditure, human resources status, and other multi-dimensional information. These data are collected based on in-depth market research and financial analysis, aiming to give a comprehensive picture of the enterprise’s financial health and potential operational risk. The database has been widely used in academic research in many related fields, and the validity and reliability of its data have been widely verified. By using this database, a relatively comprehensive and representative sample set can be obtained for evaluating the operational risk early warning model. The data for corporate bankruptcy prediction is sourced from the Corporate Bankruptcy Prediction database, available at: https://www.kaggle.com/datasets/shuvamjoy34/us-bankruptcy-prediction-data-set-19712017. This database encompasses a large number of financial indicators and corporate basic information, aiming to predict whether an enterprise is likely to go bankrupt by analyzing these data. The database includes various financial ratios and performance indicators, covering aspects such as an enterprise’s profitability, asset management, debt situation, and liquidity. These indicators are selected based on extensive financial theory and empirical research, providing a comprehensive reflection of corporate financial health and operational risks.

In the data preprocessing stage, the FCM clustering algorithm is used to classify the original data. The FCM algorithm excels in handling complex and fuzzy data. Unlike traditional hard clustering methods, such as the K-means algorithm, FCM employs a soft clustering approach, where each data point is not only assigned to one category but is instead assigned a degree of membership for each category. This allows FCM to maintain good classification performance even when there is overlap or uncertainty between data points [29]. Furthermore, the clustering results of FCM exhibit good robustness, enabling effective handling of noisy and missing data, thereby enhancing the model’s prediction accuracy. In the assessment of corporate operational risks, the FCM algorithm can accurately distinguish between different risk categories, particularly when dealing with multidimensional data, demonstrating stronger classification capability than traditional clustering methods [30]. The FCM algorithm can divide the data into several clusters based on the intrinsic characteristics of the data, and the data within each cluster has a high similarity, while the data between different clusters has a large difference. This feature enables the FCM algorithm to effectively extract useful information from the original data and reduce noise and redundancy in the data, thereby improving the quality and efficiency of subsequent analysis.

The FCM clustering algorithm allocates data points by membership value, which can better deal with the overlap and uncertainty between data points, and improve the accuracy and robustness of classification [31]. In the corporate operational risk data, there may be overlapping areas between different risk types, and the FCM clustering algorithm can effectively handle this situation.

Market, R&D, human resource, and financial risks are selected as the main indicators to evaluate enterprise risk, which are determined based on in-depth analysis of enterprise bankruptcy cases and existing literature. Market risk reflects the pressure and uncertainty of market competition faced by enterprises. R&D risk is closely related to the technological innovation ability and future competitiveness of enterprises. The financial risk is directly related to the capital structure and solvency of the enterprise. The human resource risk focuses on the human resource management and employee stability of the enterprise. These four risk factors are interrelated and influence each other, which together constitute an important part of the overall operational risk of the enterprise.

### 3.3. Risk indicator calculation based on the CRITIC weighting method

The calculation using the CRITIC weighting method begins with standardizing the data to eliminate the effects of different measurement units and value ranges among the indicators. The standardization process can be written as Eq. (1):


$$Y_{ij}=\frac{Y_{i}-min\left(Y_{i}\right)}{\max\left(Y_{i}\right)-min\left(Y_{i}\right)}$$ (1)

$Y_{ij}$ represents the standardized data; $Y_{i}$ denotes the raw data; $\max\left(Y_{i}\right)$ and $min\left(Y_{i}\right)$ refer to the maximum and minimum values of the *i*-th indicator, respectively. To calculate data volatility, i.e., the standard deviation $S_{j}$ of each indicator, Eq. (2) is used:


$$S_{j}=\sqrt{\frac{\sum_{i=1}^{n}{\left(Y_{ij}-\bar{Y_{j}}\right)}^{2}}{n-1}}$$ (2)

$\bar{Y_{j}}$ refers to the mean value of the *j*-th indicator, and *n* denotes the sample size. To calculate data correlation, the inverse of the Pearson correlation coefficient is used, as represented in Eq. (3):


$$R_{j}=\frac{1}{1-r_{ij}}$$ (3)

$r_{ij}$ means the Pearson correlation coefficient between indicators *i* and *j*. To calculate the information amount $C_{j}$ for each indicator, Eq. (4) is applied:


$$C_{j}=R_{j}\times S_{j}$$ (4)

$R_{j}$ and $S_{j}$ represent data correlation and data volatility. Finally, the CRITIC weight $W_{j}$ for each indicator is determined using Eq. (5):


$$W_{j}=\frac{C_{j}}{\sum_{k=1}^{m}C_{k}}$$ (5)

*m* is the total number of indicators.

This study selects 15 primary assessment indicators, including the revenue growth rate, cash ratio, debt-to-equity ratio, and R&D expenditure, among others. These indicators comprehensively reflect the market, R&D, financial, and human resource risks of an enterprise. The CRITIC method is utilized to calculate the weight of each indicator to determine its relative importance in the overall risk assessment. The CRITIC method determines weights by calculating the correlation between indicators and their standard deviation, ensuring the model can more accurately capture risk characteristics. These weighted indicators are fed into the FCM clustering algorithm and RF model as input features. The FCM clustering algorithm is used for data preprocessing and classification, while the RF model is employed for the final risk prediction.

### 3.4. Implementation of the FCM clustering algorithm

Operational risk early warning often faces the problem of incomplete data labels or high acquisition costs. As an unsupervised learning algorithm, FCM can cluster based on the characteristics of the data itself, effectively distinguish enterprise samples with diverse risk levels, and provide more targeted data subsets for subsequent supervised learning. Enterprise operational risk involves multiple dimensions and levels of data, including financial indicators, market data, and operational data. FCM can process these multidimensional data by calculating the similarity between the samples and dividing them into different clusters to reveal the underlying structure and pattern in the data.

In corporate operational risk data, there may be overlapping areas between different types of risks. FCM, through its membership values, can better handle such situations, enhancing the accuracy and robustness of classification. The fuzzifier coefficient *m* of FCM can be adjusted according to specific application scenarios, offering greater flexibility and the ability to adapt to different data distribution characteristics. In contrast, K-means struggles to effectively handle overlapping areas between different risk types, which can lead to misclassification. DBSCAN has two critical parameters—the neighborhood radius ε and the minimum number of points MinPts. The selection of these parameters significantly impacts the clustering results, but choosing the appropriate parameters can be challenging.

FCM is an efficient clustering technique for data classification and pattern recognition. The core of the FCM algorithm is an iterative optimization process that partitions data points into a predetermined number of clusters, maximizing similarity within clusters and differences between clusters. This is achieved by minimizing a specific objective function that measures the membership and distance between sample points and cluster centers. The FCM algorithm is utilized to process and analyze corporate operational risk data. The objective function of the FCM algorithm is defined in Eq. (6):


$$J_{m}=\sum_{i=1}^{N}\sum_{j=1}^{m}ij_{m}^{u}\cdot d{\left(x_{i},c_{j}\right)}^{2}$$ (6)

**Fig 2:**
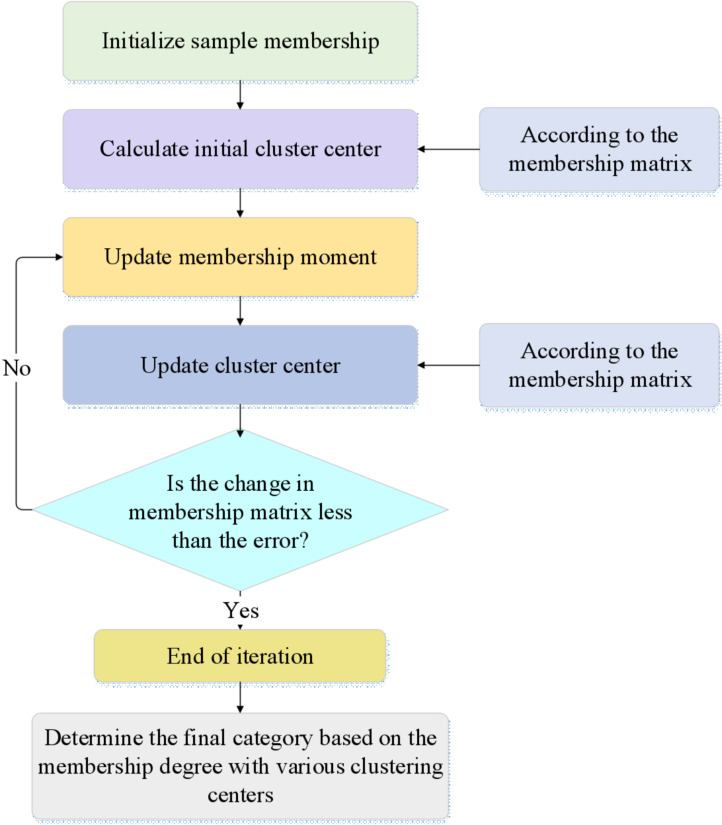
The construction process of the FCM clustering algorithm.

$J_{m}$ refers to the objective function; *m* indicates the number of clusters; *N* stands for the total number of samples; $c_{j}$ means the *j* -th cluster center; $x_{i}$ represents the *i* -th sample; $u_{ij}$ is the membership degree of sample $x_{i}$ to cluster center $c_{j}$; $d\left(x_{i},c_{j}\right)$ denotes the distance between sample $x_{i}$ and cluster center $c_{j}$. The construction process of the FCM clustering algorithm is displayed in Fig 2:

In Fig 2, the number of clusters *m* is first determined, and the membership matrix is randomly initialized. The membership matrix is an N×m matrix where each element’s value ranges between 0 and 1, and the sum of elements in each row equals 1. Based on the current membership matrix, the positions of each cluster center are recalculated. The new position of cluster center $c_{j}$ is shown in Eq. (7):


$$c_{j}=\frac{\sum_{i=1}^{N}ij_{m}^{u}\cdot x_{i}}{\sum_{i=1}^{N}ij_{m}^{u}}$$ (7)

Using the updated cluster centers, the membership degree of each sample point $x_{i}$ to each cluster center $c_{j}$ is recalculated. The calculation of membership reads:


$$ij_{m}^{u}=\frac{1}{\sum_{k=1}^{m}{\left(\frac{d\left(x_{i},c_{k}\right)}{d\left(x_{i},c_{j}\right)}\right)}^{2}}$$ (8)

The iterative optimization process repeatedly applies Eqs. (7) and (8) until the change in the membership matrix falls below a predefined error threshold or the predetermined number of iterations is reached. Once the change in the membership matrix is smaller than the set threshold, the algorithm terminates, and the resulting clusters are deemed stable. Finally, the clustering results are used for the subsequent construction of the risk assessment and early warning model [32–34]. Additionally, this study employs the silhouette coefficient and the Davies-Bouldin Index (DBI) to evaluate the performance of the FCM clustering algorithm [35]. By calculating the silhouette coefficient for each sample, the experiment provides an intuitive understanding of each cluster’s cohesion and separation. Simultaneously, the overall clustering performance is assessed using the DBI index. The silhouette coefficient, which ranges from −1 to 1, assesses the rationality of the clustering for each data point. It considers both the similarity of a data point to other points within the same cluster (cohesion) and its dissimilarity to points in the nearest cluster (separation). The calculation of the silhouette coefficient is expressed in Eq. (9):


$$s\left(x_{m}\right)=\frac{b\left(x_{m}\right)-a\left(x_{m}\right)}{max\left\{a\left(x_{m}\right),b\left(x_{m}\right)\right\}}$$ (9)

$s\left(x_{m}\right)$ means the silhouette coefficient value of data point $x_{m}$, $a\left(x_{m}\right)$ represents the average distance from $x_{m}$ to other points within its cluster (intra-cluster dissimilarity), and $b\left(x_{m}\right)$ refers to the average distance from $x_{m}$ to points in the nearest other clusters (nearest-cluster distance). The DBI index is another metric to assess clustering performance, where a lower value indicates better clustering results. This index considers intra-cluster similarity and inter-cluster dissimilarity. The calculation of the DBI index is as follows:


$$R_{ij}=\frac{avg\left(C_{i}\right)+avg\left(C_{j}\right)}{d_{cen}\left(C_{i},C_{j}\right)}$$ (10)

$$DBI=\frac{1}{k}\sum_{i=1}^{k}\underset{\;j\neq i}{\underset{︸}{max}}\;R_{ij}$$ (11)

$R_{ij}$ and $d_{cen}\left(C_{i},C_{j}\right)$ represent the relative scatter and centroid distance between clusters $C_{i}$ and $C_{j}$; $avg\left(C_{i}\right)$ and $avg\left(C_{j}\right)$ respectively denote the average scatter of clusters $C_{i}$ and $C_{j}$; *k* refers to the total number of clusters, and the summation symbol across all clusters computes the maximum $R_{ij}$ value for each cluster, followed by averaging them.

The K-fold cross-validation method is employed to assess the stability and generalization ability of the model. Specifically, the original dataset is divided into K subsets, with one subset retained as the validation set and the remaining K-1 subsets used as the training set for each iteration. This process is repeated K times, with each subset serving exactly once as the validation set. By employing this method, a more comprehensive evaluation of the model’s performance across different data subsets can be achieved, thus reducing the impact of random errors and enhancing the model’s generalization ability.

To prevent the model from becoming overly complex and thus overfitting, regularization techniques are introduced in the RF model in this study. Regularization limits the complexity of the model by adding a penalty term to the loss function, preventing the model from overlearning noise in the training data. In this study, model complexity is controlled by managing parameters such as the maximum depth of the decision tree and the minimum number of sample segments. Additionally, a minimum number of samples required at the leaf nodes is set to ensure that the model does not lose its generalization ability due to overfitting.

To find the optimal combination of model parameters, this study utilizes grid search. Grid search is an exhaustive search method that evaluates the model’s performance for all possible combinations within a predefined parameter space, combined with cross-validation techniques. The set of parameters that yield the best performance is ultimately selected as the optimal configuration for the model. This study focuses on tuning two critical parameters. The maximum depth controls the complexity of individual decision trees and prevents overfitting; The maximum number of features determines the number of features considered at each decision node, affecting the diversity of the trees and the model’s generalization ability.

### 3.5. Optimization of RF algorithm parameters

**Fig 3:**
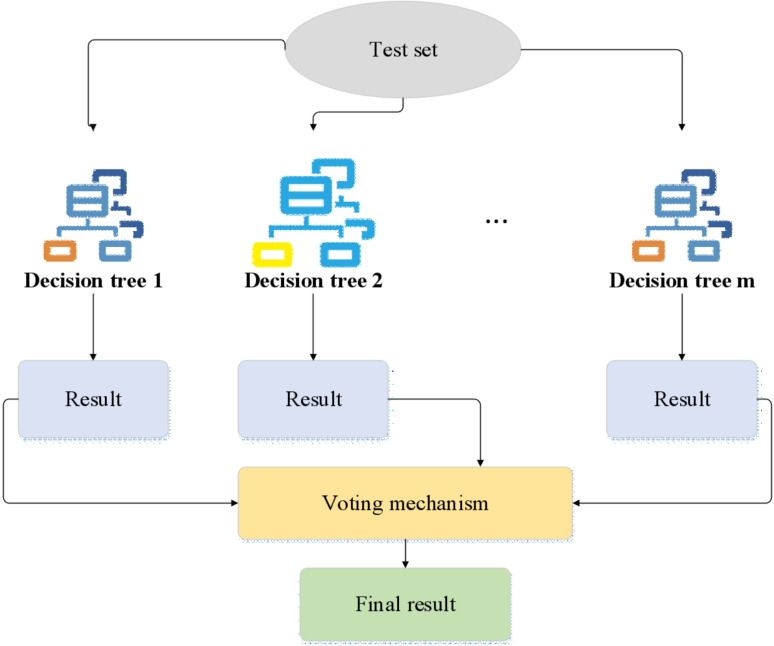
Construction process of RF.

RF can improve the prediction accuracy of the model by constructing the integration of multiple decision trees. In addition, it can handle high-dimensional data and complex nonlinear relations, which is very suitable for the multi-dimensional and multi-factor risk assessment scenario of enterprise operational risk. Although RF is an integrated learning model, its construction process of an internal decision tree is relatively intuitive. Moreover, the model’s prediction results can be interpreted by indicators such as the importance of features, thus offering a decision basis for enterprise managers. RF is an ensemble learning method that addresses classification and regression problems by constructing multiple decision trees. It has garnered widespread attention due to its effectiveness in anomaly detection and prediction tasks. Compared to traditional single decision tree models, RF enhances overall model accuracy and robustness by aggregating predictions from multiple decision trees [36]. The construction process of RF is illustrated in Fig 3:

Fig 3 illustrates the construction process of an RF model. Initially, samples are randomly drawn with replacements from the original training dataset to form multiple distinct training subsets. During the training of each decision tree, a random subset of features is selected, and the tree is constructed based on these features and the corresponding sample data. Each tree is independently trained using its designated samples and features until a predefined tree depth or other stopping criteria is met. The prediction from each tree results in a probability distribution. The final prediction of the RF model is derived by averaging the weighted probabilities of all individual trees’ predictions, with the category having the highest weighted probability selected as the final prediction.

This study particularly focuses on parameter tuning of the RF algorithm. Parameter tuning is a crucial step in improving model performance, and the experiment employs Grid Search to find the optimal model parameters. Grid Search evaluates the performance of different parameter combinations through cross-validation, thereby determining the best parameter settings. In RF, two of the most important parameters are:

(i) Maximum Depth of Trees: The complexity of each tree is controlled to prevent overfitting.
(ii) Maximum Number of Features Considered for Splitting: The number of features considered at each decision node is determined, affecting the diversity of trees and the model’s generalization capability.

This study achieves optimal model prediction performance by using Grid Search for meticulous tuning. Gini impurity, which measures node impurity in decision tree splitting, is calculated as Eq. (12):


$$G\left(p\right)=\sum_{k=1}^{n}p_{k}\left(1-p_{k}\right)$$ (12)

$p_{k}$ represents the proportion of samples belonging to the *k* -th class. Ultimately, the classification result of the RF algorithm is depicted as Eq. (13):


$$H\left(x\right)=argmax_{y}\sum_{i=1}^{m}I\left(h_{i}\left(x\right)=y\right)$$ (13)

$H\left(x\right)$ denotes the prediction result of the RF model; $h_{i}$ refers to the prediction of each decision tree; *I* represents the indicator function; *y* means the class label.

Parameter optimization is a crucial part of constructing and tuning machine learning models and is often considered part of standard procedures. However, in this study, the optimal combination of parameters found through a refined grid search approach plays a key role in improving the RF model’s performance in corporate operational risk early warning systems. Parameter optimization can enhance the model’s prediction accuracy and improve its stability and generalization ability, enabling this model to make more reliable and accurate judgments in the face of unknown data. The RF algorithm’s parameter settings after parameter optimization are outlined in Table 1. After grid search, this study determined the following parameter settings. The number of trees in the forest is 200, the maximum depth is 30, the minimum sample required for internal node segmentation and leaf nodes is 5 and 2, and the number of random seeds is 42.

**Table 1:** The parameter settings of the RF algorithm after parameter optimization.

Name of parameter	Description	Setting Value
n_estimators	The number of trees in a random forest	200
max_depth	The maximum depth of a single decision tree	30
max_features	The number of features considered at each decision node	sqrt
min_samples_split	Minimum number of samples required for internal node segmentation	5
min_samples_leaf	Minimum number of samples required for leaf nodes	2
bootstrap	Whether to use guidance samples	True
oob_score	Whether to use out-of-bag samples to evaluate the generalization error of the model	True
class_weight	Category weight	None
random_state	Random seeds can ensure the reproducibility of results	42

Data preprocessing is a key step to ensure the quality of model input data. Data truncation is a common problem in operational risk management, which may lead to deviation from risk assessment results [37]. Extreme values (such as data points with a Z-score greater than 3 or less than −3) are replaced with the nearest non-extreme value to reduce the impact of noise and outliers in the data on the model. To effectively address this problem, the truncation point in the dataset is first identified, namely, the reporting threshold set by the state or institution (€2,000). Subsequently, statistical methods are employed to quantify the effect of truncation on the overall distribution of the dataset.

### 3.6. Design of experiment

This study implements the FCM clustering and RF model using the Python programming language and the Scikit-learn library. The specific code and data processing scripts have been uploaded to GitHub.

To evaluate the performance of the proposed FCM-RF model under different conditions, this study designs three distinct experimental setups. These setups aim to explore the impact of different asset allocations and adjustment frequencies on model performance, thereby guiding practical applications.

#### Experimental setup 1.

Asset allocation ratio: 20% for stocks, 60% for bonds, and 20% for cash and equivalents. Adjustment frequency of investment portfolio: It is adjusted annually; Stop-loss point: A stop-loss is triggered when the stock investment loss reaches 10%; Dynamic rebalancing: The portfolio is reviewed quarterly and dynamically rebalanced according to the preset asset allocation ratios.

#### Experimental setup 2.

Asset allocation ratio: 30% for stocks, 50% for bonds, and 20% for cash and equivalents; Adjustment frequency of investment portfolio: It is adjusted once every quarter; Stop-Profit point: When the profit from stock investment reaches 15%, partial selling can be considered.

#### Experimental setup 3.

Asset allocation ratio: 40% for stocks, 40%for bonds, and 20% for cash and equivalents; Adjustment frequency of investment portfolio: It is adjusted once a month; Stop-Profit point: When the stock investment loss reaches 10%, the stop loss is triggered.

## 4. Results and discussions

### 4.1. CRITIC weight calculation results for risk indicators

**Fig 4:**
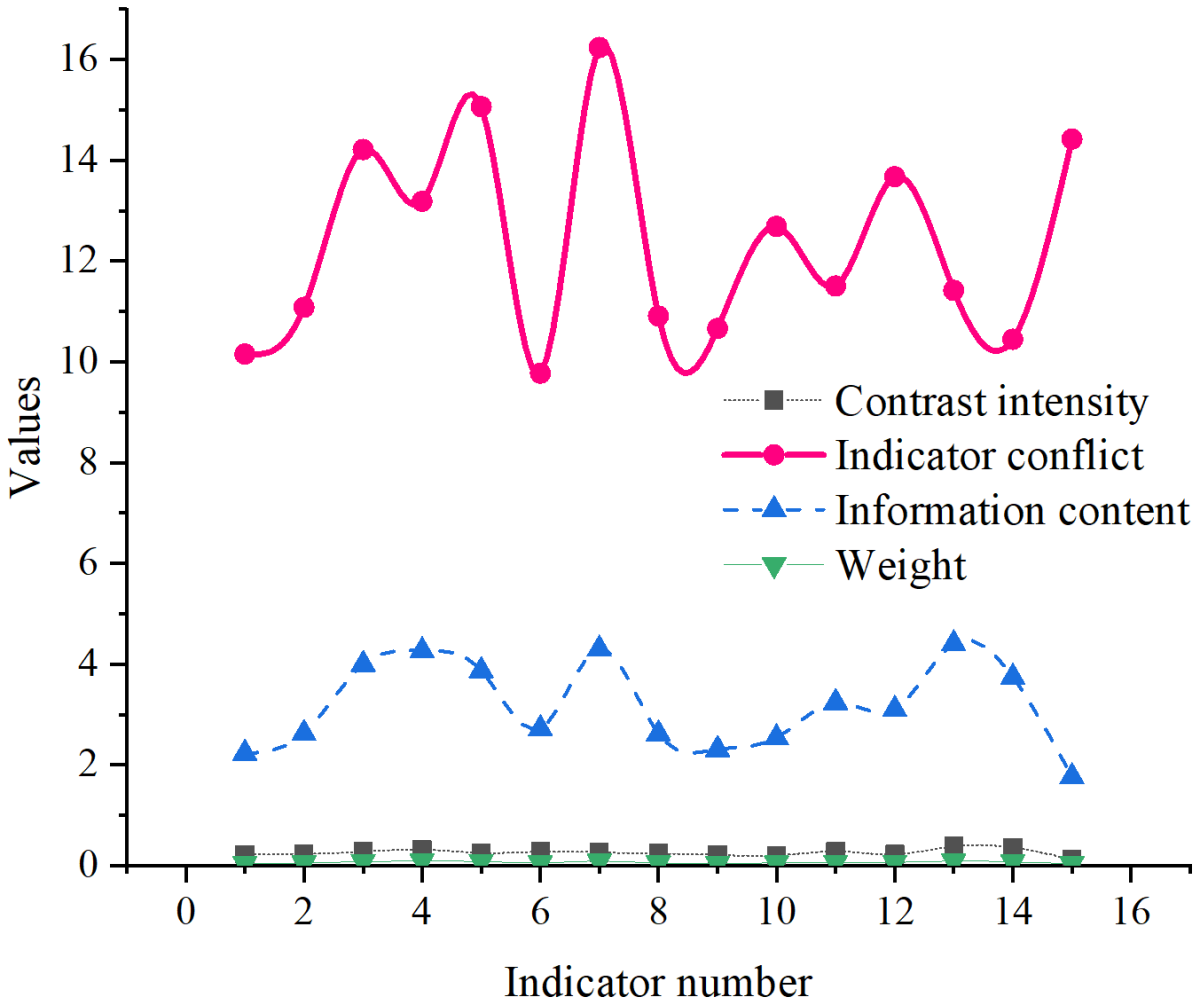
CRITIC weight calculation results for corporate operational risk warning indicators.

The CRITIC weight calculation results for corporate operational risk warning indicators are denoted in Fig 4. Indicator 13 (Debt-to-Equity Ratio) has the highest weight of 0.09, indicating its crucial importance in corporate operational risk warning. The Debt-to-Equity Ratio reflects the enterprise’s financial leverage and debt repayment capability. A higher weight signifies that debt level is a key factor in assessing enterprise risk. Indicators 7 and 3 (Total Asset Turnover Ratio and Cash Ratio) have weights of 0.09 and 0.08, respectively. These two indicators are closely related to the corporate cash flow situation, emphasizing the significance of cash flow in risk warnings. Additionally, the weights of indicators 2 (Net Profit Margin on Sales) and 4 (Net Profit Margin on Total Assets) are relatively high, at 0.05 and 0.09, respectively. These indicators reflect corporate profitability, highlighting profitability as another important dimension in assessing its operational risk. Indicators 1 (Revenue Growth Rate) and 5 (R&D Expenditure as a Percentage of Revenue) have lower weights of 0.05 and 0.08, respectively. This indicates that within the current evaluation framework, the role of revenue growth and the proportion of R&D expenditure to revenue in risk warning is relatively small compared to other indicators.

Financial indicators are primarily used to assess the financial health and financial risks of enterprises. These indicators, calculated from financial statement data, comprehensively reflect the profitability, debt-paying ability, asset management, and capital usage efficiency of an enterprise. The weight values for the revenue growth rate, cash ratio, debt-to-equity ratio, net profit margin, total asset turnover ratio, fixed asset utilization rate, return on capital, and debt maturity structure are 0.12, 0.10, 0.09, 0.04, 0.04, 0.03, 0.03, and 0.02, respectively. Higher weight values indicate the significance of these indicators in risk assessment. The weight values for market share, customer satisfaction, supplier dependency, accounts receivable turnover, and inventory turnover are 0.06, 0.06, 0.05, 0.05, and 0.05, respectively. Higher weight values illustrate the importance of these indicators in market risk assessment.

The weight values for employee satisfaction and talent turnover rate are 0.07 and 0.05. These indicators, calculated from employee surveys and personnel data, reflect the satisfaction and loyalty of employees, and the enterprise’s talent retention capabilities. The weight values for R&D expenditure and debt maturity structure are 0.08 and 0.02, primarily used to assess the enterprise’s technological innovation capabilities and debt structure.

Overall, the data reflects the relative importance of different indicators in the corporate operational risk early warning system. In terms of weight values, indicators such as “R&D personnel proportion,” “R&D investment ratio,” “capitalized R&D investment ratio,” and “Annual incentive plans” have a significant impact on corporate operational risks. Moreover, some indicators, such as “proportion of management personnel,” play a smaller role in this model, likely contributing less to the risk early warning process.

### 4.2. FCM algorithm operational risk clustering results

**Fig 5:**
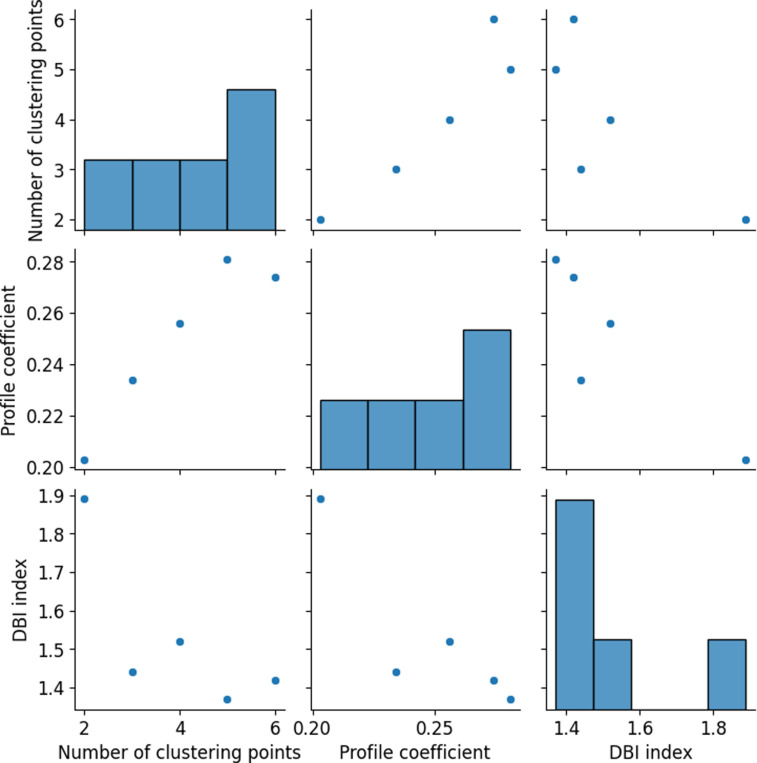
Clustering evaluation results of the FCM algorithm.

Fig 5 presents the clustering evaluation results of the FCM algorithm, illustrating the variations in silhouette coefficient and DBI as the number of clusters increases. When the number of clusters is 5, the FCM algorithm achieves optimal clustering performance, with the highest silhouette coefficient and the lowest DBI. This suggests a well-balanced cohesion within clusters and separation between clusters. Partitioning the data into 5 clusters effectively captures the underlying structure of the data while maintaining high intra-cluster similarity and substantial inter-cluster dissimilarity. Therefore, selecting 5 clusters for corporate operational risk warning facilitates more precise classification and assessment of operational risks.

**Fig 6:**
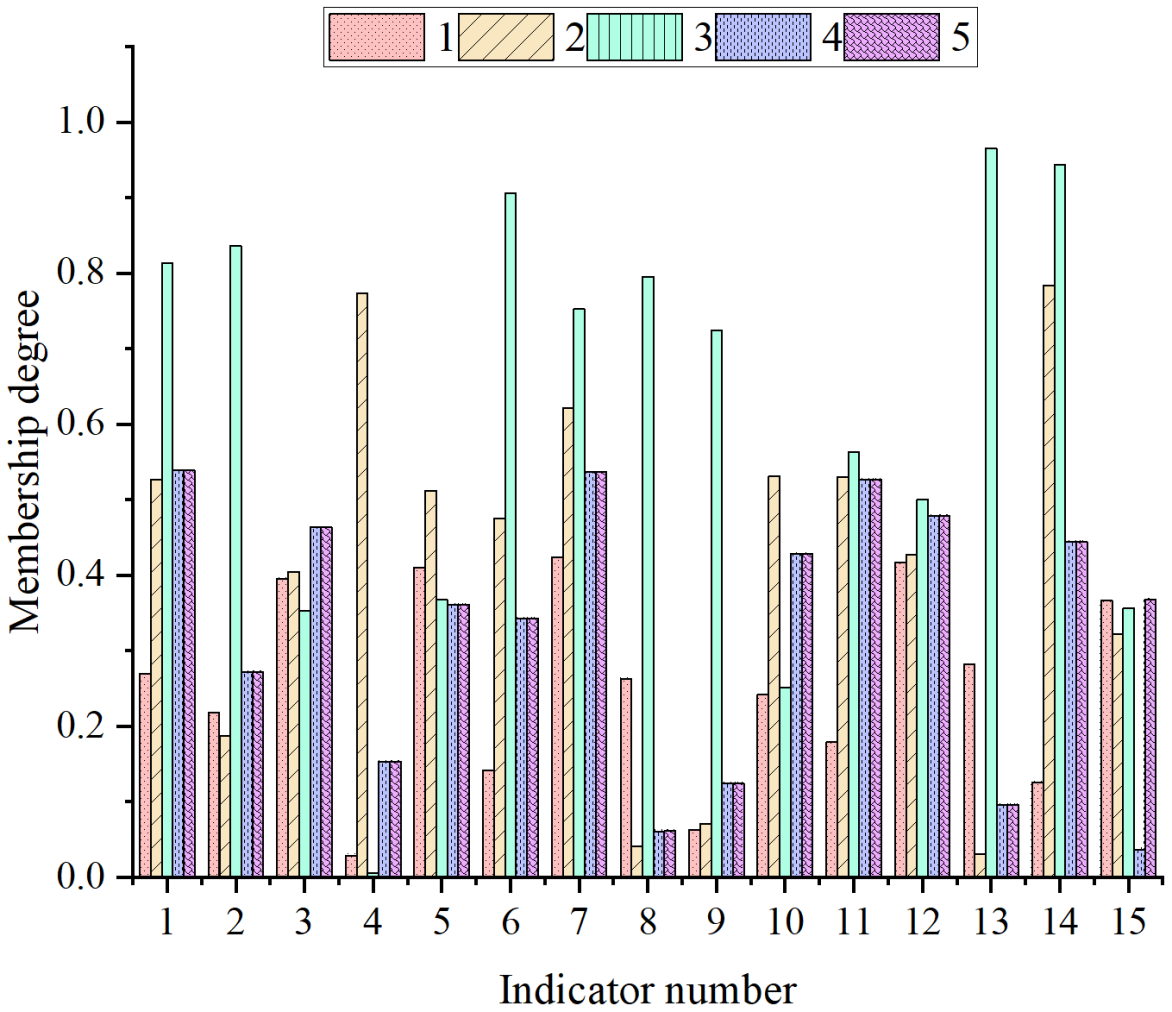
The membership distribution of various indicators in different risk categories by the FCM algorithm (Note: Clustering categories (Medium risk (2, 3)/ High risk (5)/ Risk-free (1)/ Low risk (4)).

Fig 6 presents the membership distribution of various indicators across different risk categories by the FCM algorithm. Based on the data in Fig 6, the distribution of each indicator across different risk categories can be analyzed. Each row of the data represents a different classification value of a corporate operational risk indicator, including the indicator number, the values of five indicators, and the risk category to which the indicator belongs (e.g., low risk, medium risk, high risk). This data reveals the distribution of each indicator across different risk categories (such as medium, low, and high risks). First, the indicators in the “medium risk” category are examined. Most indicators fall into the medium-risk category, indicating that these indicators are more often within the medium-risk range when assessing corporate operational risks. For instance, the first indicator (Revenue Growth Rate) and the second indicator (Net Profit Margin from Sales) have values of 0.27 and 0.53, respectively, both of which fall into the “medium risk” category. Furthermore, the third indicator (Accounts Receivable Turnover Ratio) and the seventh indicator (R&D Personnel Proportion) also belong to the medium-risk category. This reflects a balanced role in the operational risk early warning system, as they are neither too high nor too low, and are positioned at a medium-risk level. Next, the indicators in the “high risk” category are analyzed. There are fewer indicators in the high-risk category, but they carry greater weight in the risk evaluation. For example, the fifth indicator (R&D Investment to Operating Income Ratio) and the sixth indicator (R&D Personnel Proportion) are classified as high risk. This suggests that these indicators have higher values in risk assessments, reflecting a higher level of corporate operational risk. Specifically, the sixth indicator, “R&D Personnel Proportion,” with a value of 0.91, stands out as exhibiting a prominent high-risk characteristic among the five indicators. In the “low risk” category, the fourth indicator (Capitalized R&D Investment Ratio) and the eighth indicator (Net Profit Margin on Total Assets) are classified as low risk. This indicates that these indicators demonstrate relatively stable and low-risk characteristics within the enterprise. Similarly, the ninth indicator (Net Asset Growth Rate) and the fifteenth indicator (Management Personnel Proportion) also fall into the low-risk category. This shows that these two indicators do not exhibit significant volatility or risk during the analysis. Lastly, the data reveals the sensitivity of different risk categories to various risk factors in corporate operations, reflecting the volatility of these indicators under different risk levels. The medium-risk indicators are more common, suggesting that these indicators tend to remain at a medium-risk level for most enterprises. In contrast, high-risk and low-risk indicators are relatively fewer, indicating that the risks associated with these indicators may be more extreme. In evaluating enterprise operational risks, combining these risk category classifications can help predict and prevent potential operational risks more accurately.

**Fig 7:**
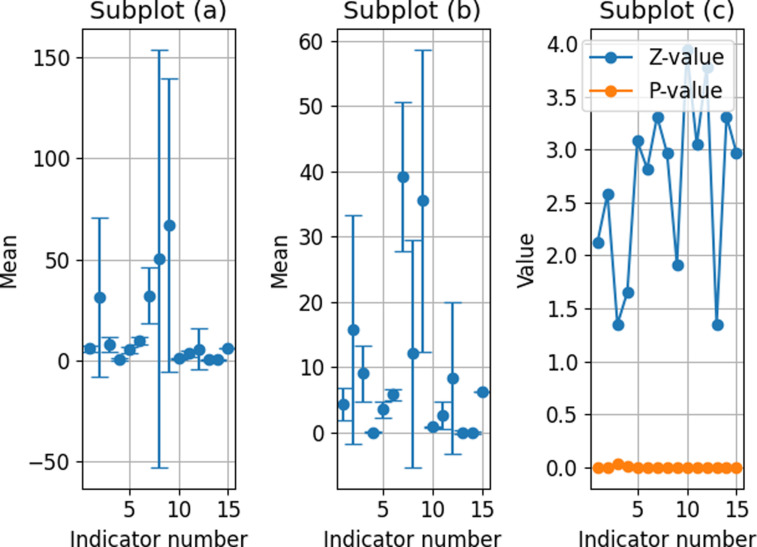
Corporate operational risk indicator statistical differences results.

The statistical differences in corporate operational risk indicators are plotted in Fig 7. Observing (a) normal points and (b) risk points in terms of mean and standard deviation, it is evident that for most indicators, the mean of risk points is lower than that of normal. This suggests that these indicators perform worse than normal when enterprises are in a risky state. Additionally, the standard deviation of risk points is generally higher than that of normal points, indicating greater variability in data under risky conditions and an increase in uncertainty and instability faced by the enterprise. Specifically, the mean of risk points for indicators 1, 2, 5, 7, 10, 11, and 12 is markedly lower than the normal point, and the standard deviation of risk points for these indicators is also higher, illustrating a greater decrease and variability under risk conditions. Particularly for indicators 1 and 10, their z-values and p-values in the two-sample Kolmogorov-Smirnov test exhibit extreme differences, with z-values far greater than 0 and p-values of 0, demonstrating significant differences in the distributions between normal and risk points. The basic assumption of the KS test is that the sample data is independent and equally distributed and does not depend on any particular parameter form. The calculation of the z value is based on the difference between the cumulative distribution function (CDF) of the two samples, specifically by calculating the maximum vertical distance between the two CDFs at all possible points. This maximum distance is the z-value, which reflects the maximum degree of difference between the two sample distributions. When the z-value is large and the corresponding p-value is less than the significance level (0.05), the null hypothesis can be rejected and the two samples are considered to be from different distributions.

In addition, although the mean risk points of indicators 3, 4, 6, 8, and 9 are lower than normal, the differences are not as significant as other indicators. Indicators 3 and 8 also show statistical significance in z-values and p-values, but their increased variability under risky conditions is not as pronounced as for some other indicators. There is not much difference in the mean values of indicators 13, 14, and 15 between risk and normal points, but their standard deviations have increased between risk points. This suggests that although the average levels of these indicators do not decrease much under risky conditions, they face greater variability. Particularly for indicator 14, with a z-value of 3.311 and a p-value of 0 in the two-sample Kolmogorov-Smirnov test, significant differences between normal and risk points are indicated.

Fig 7a and 7b respectively display the mean and standard deviation of operational risk indicators for normal and risky points. By comparing these two subplots, it can be observed that under risky conditions, the mean of most indicators is lower than under normal conditions, indicating that these indicators perform poorly when the enterprise is facing risks. The standard deviation of risky points is generally higher than that of normal points, demonstrating an increase in data variability under risky conditions, with increased uncertainty and instability faced by the enterprise. In this experiment, several sets of experiments are designed to test the model’s performance under diverse parameter settings. These parameters include but are not limited to, the weight allocation of investment strategies, trading frequency, risk management thresholds, etc. Each group of experiments is run independently several times to ensure the stability and reliability of the results.

Parameter setting 1: (1) Asset allocation ratio: 60% for stocks, 30% for bonds, and 10% for cash and equivalents. This setting tends towards a more aggressive investment strategy, aiming to obtain higher potential returns through higher stock allocation, but also assumes higher market risks. (2) Trading frequency: The investment portfolio is adjusted once a month. (3) Stop-loss point: When the stock investment loss reaches 10%, the stop loss is triggered.

Parameter setting 2: (1) Asset allocation ratio: 40% for stocks, 40% for bonds, and 20% for cash and equivalents. This setting is more balanced and aims to achieve a better balance between returns and risks. A balanced allocation of stocks and bonds helps to diversify risk, while a higher allocation of cash and equivalents provides liquidity. (2) Trading frequency: The investment portfolio is adjusted once every quarter. (3) Stop-Profit point: When the profit from stock investment reaches 15%, partial selling can be considered.

Parameter setting 3: (1) Asset allocation ratio: 20% for stocks, 60% for bonds, and 20% for cash and equivalents. This setting tends towards a conservative investment strategy, reducing overall risk through higher bond allocation and pursuing stable returns. (2) Trading frequency: The investment portfolio is adjusted once a year. (3) Dynamic rebalance: The investment portfolio is checked once every quarter and dynamically rebalanced according to the preset asset allocation ratio.

Table 2 shows the calculation results of the mean, standard deviation, and reward-risk ratio of the model under different parameter settings. It can be found that while Setting 3 has the highest mean (6.5%), it also has the largest standard deviation (3.0%), resulting in a relatively low reward-risk ratio (2.17). This suggests that Setting 3 is taking on a high level of risk while pursuing high returns. In contrast, Setting 2 has a slightly lower mean than Setting 3, but has a smaller standard deviation and therefore the highest reward-risk ratio (2.67). This suggests that Setting 2 strikes a better balance between risk and benefit.

**Table 2:** Model performance comparison under different parameter settings.

Number of parameter settings	Mean (%)	Standard deviation (%)	Reward-risk ratio
Setting 1	5.2	2.1	2.48
Setting 2	4.8	1.8	2.67
Setting 3	6.5	3.0	2.17

### 4.3. Predictive results of the RF model improved by FCM clustering

**Fig 8:**
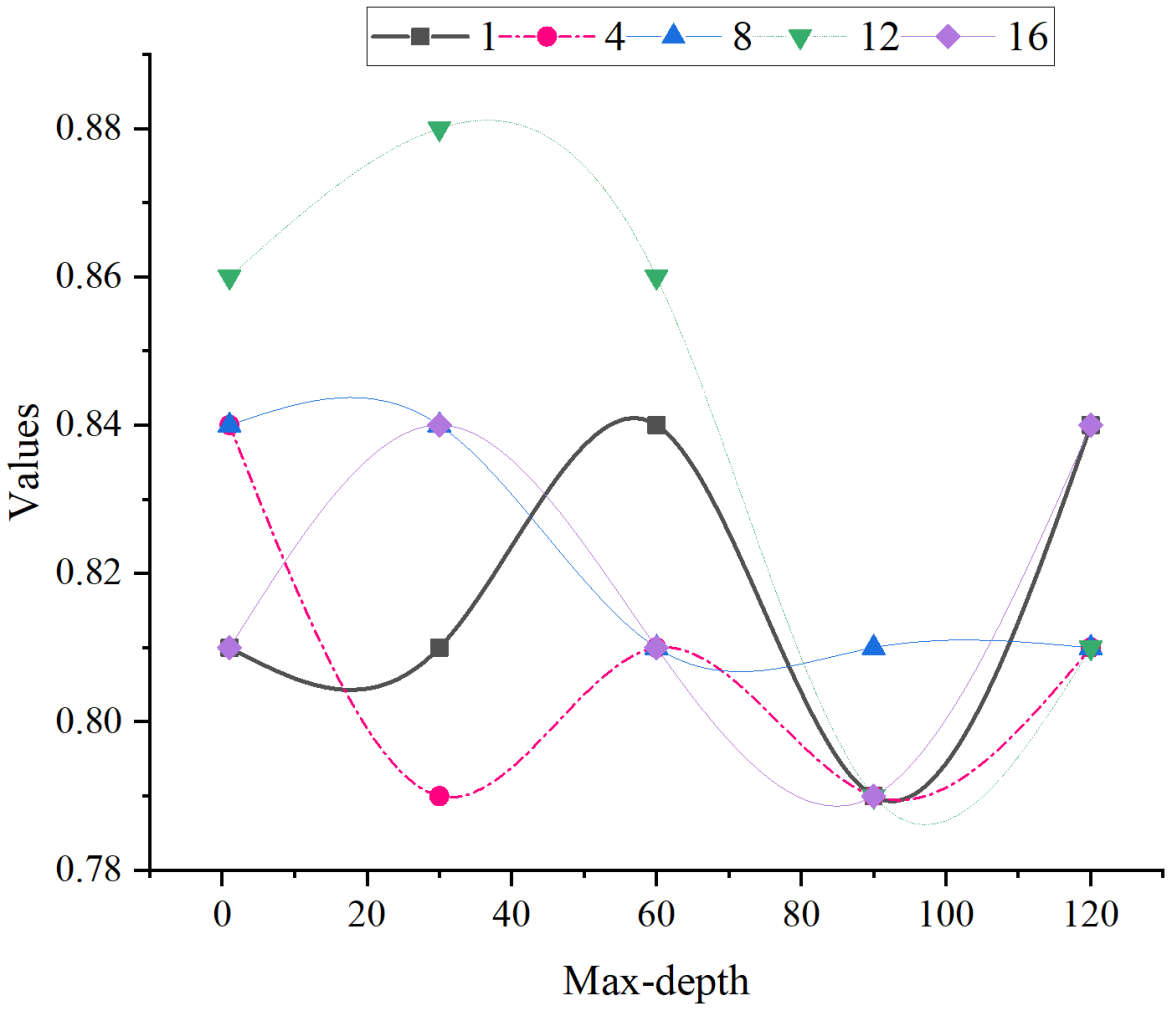
Parameter optimization results of the RF model improved by FCM clustering.

The parameter optimization results of the RF model improved by FCM clustering are suggested in Fig 8. Overall, the evaluation results of the model remain relatively stable with minimal fluctuations as the maximum depth value changes. When the maximum depth is set to 1, 30, 60, 90, and 120, the model’s evaluation results fluctuate between approximately 0.79 and 0.88. This suggests that the impact of different depth settings on model optimization is limited, and the model performs consistently across most depths. Specifically, when the maximum depth is 30, the evaluation results are the best among the four depth values, ranging from 0.81, 0.79, 0.84, 0.88, to 0.84. The highest result of 0.88 occurs at a depth of 12, indicating that the model performs optimally at this depth. This implies that setting the maximum depth to 30 yields better optimization of the model, particularly at a depth of 12. In contrast, when the maximum depth is set to 1, 60, 90, or 120, the model’s evaluation results are relatively similar. It typically fluctuates between 0.79 and 0.84, showing relatively stable performance without significant improvements or declines. When the maximum depth is 1, the model’s evaluation results are lower, possibly due to the limited learning capacity of the model caused by the shallow tree depth. Generally, based on the data, for the RF model improved by FCM clustering, selecting up to 12 features and setting the maximum tree depth to 30 can achieve optimal model performance.

**Fig 9:**
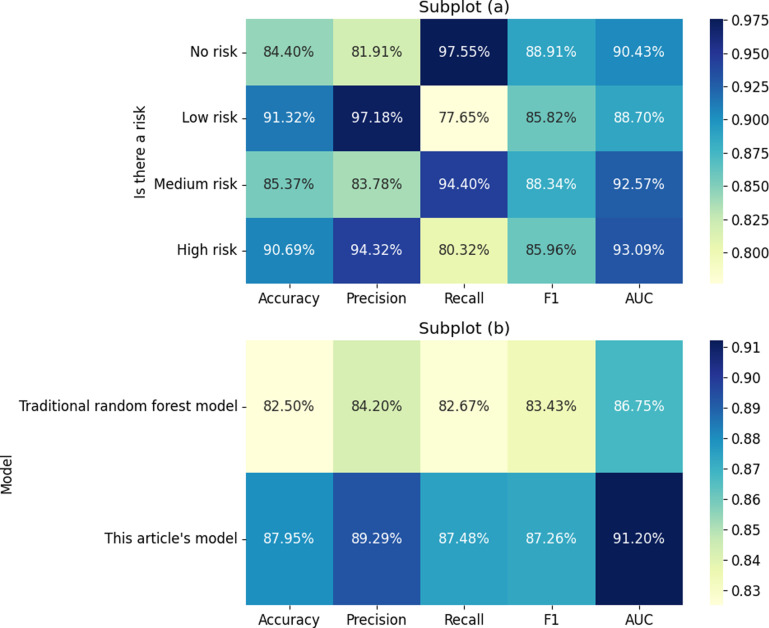
Risk prediction results of the RF model improved by FCM clustering ((a) Different risk prediction results; (b) Prediction results of different models).

The risk prediction results of the RF model improved by FCM clustering are presented in Fig 9. According to the data, the RF model improved by FCM clustering developed in this study outperforms the traditional RF model across multiple performance evaluation metrics. Specifically, the model achieves an accuracy of 87.95%, an Area Under the Curve (AUC) of 91.20%, a recall of 87.48%, a precision of 89.29%, and an F1 score of 87.26%. Compared to the traditional RF model, these metrics represent improvements of 6.45%, 4.45%, 5.09%, 4.81%, and 3.83%, respectively. Fig 9a shows the parameter optimization results of the RF model improved by FCM clustering. According to the data, the model performance can be optimized by selecting up to 12 features and setting the maximum tree depth to 30. The optimization of these parameters helps to improve the prediction accuracy and robustness of the model, thus more reliably assessing the corporate operational risks.

**Fig 10:**
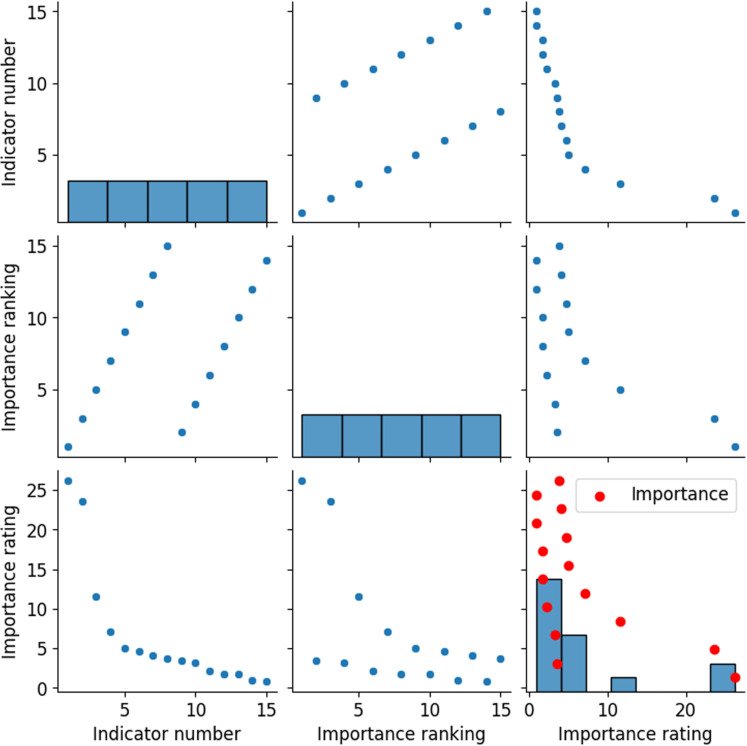
Feature importance ranking results of model risk indicators.

The ranking results of the feature importance of risk indicators for the model are depicted in Fig 10. The feature importance ranking highlights several indicators crucial for evaluating corporate operational risks. Factors such as revenue growth rate and cash ratio should be particularly emphasized in risk management. Meanwhile, profitability, liquidity, innovation capability, and financial structure are critical considerations. Fig 10 reveals the ranking of feature importance for the model’s risk indicators. The ranking results show that the revenue growth rate and cash ratio are the two most critical indicators in assessing the operational risks of an enterprise. The high ranking of these indicators emphasizes the importance of profitability, liquidity, innovation capacity, and financial structure in risk management. In the current model, the net asset growth rate, total asset turnover ratio, annual incentive plan, and the proportion of management personnel have relatively low importance scores. However, these indicators may also have a significant impact on the enterprise’s operations under certain circumstances.

### 4.4. Robustness testing of the model

Table 3 exhibits the model’s robustness test results. When the learning rate is adjusted within a reasonable range (from 0.001 to 0.1), the model’s performance fluctuates less (the accuracy changes around ± 1%), showing a low sensitivity to changes in the learning rate. When the learning rate is too high (such as 0.5), the model’s performance deteriorates significantly, indicating that the model cannot converge stably due to the high learning rate. For the regularization coefficient, the model’s performance fluctuates less when adjusted within a reasonable range (from 0.001 to 0.1). However, excessively high regularization coefficients (such as 1.0) can lead to underfitting of the model and a significant decrease in performance.

**Table 3:** The robustness test results of the model.

Parameter settings	Learning rate	Regularization coefficient	Accuracy (%)	Recall (%)	F1 score (%)
Benchmark model	0.01	0.01	85.0	80.0	82.4
Lower learning rate	0.001	0.01	84.5	79.5	81.8
Higher learning rate	0.1	0.01	85.5	81.0	83.1
Excessive learning rate	0.5	0.01	78.0	70.0	73.8
Lower regularization	0.01	0.001	85.2	80.5	82.7

To comprehensively assess the model’s robustness, this study further tests the changes in model performance under different learning rates and regularization coefficients, as well as the model’s performance on different datasets. The test results indicate that when the learning rate is adjusted between 0.001 and 0.1, the model’s performance fluctuates minimally (with accuracy changes of approximately ± 1%), showing low sensitivity to changes in the learning rate. However, when the learning rate is too high (0.5), the model’s performance remarkably declines, illustrating that the model cannot converge stably under high learning rates. Regarding the regularization coefficient, the model’s performance fluctuates minimally when adjusted within a reasonable range of 0.001 to 0.1. However, an excessively high regularization coefficient (1.0) may lead to model underfitting and a significant decrease in performance. These test results highlight the importance of selecting appropriate parameters in practical applications and guide further optimization of the model.

### 4.5. Corporate operational risk prevention and control strategies

Based on a thorough analysis of the importance of corporate operational risk warning indicators and model prediction results, it is crucial to develop effective risk prevention and control strategies. Initially, enterprises should closely monitor two key indicators: revenue growth rate and cash ratio, as they occupy the most critical positions in risk assessment. Enterprises should ensure continuous revenue growth while maintaining sufficient cash flow to address potential market fluctuations and financial needs. Subsequently, profitability is also core to risk management. Enterprises should enhance profitability by improving net profit margin and return on total assets. This can be achieved through optimizing cost structures, improving operational efficiency, and adjusting product pricing strategies. Additionally, managing the accounts receivable turnover rate and the proportion of R&D personnel is equally important. These factors are linked to the efficiency of receivables and innovation capability, crucial for sustaining competitive advantages. Moreover, attention should be given to the total asset cash recovery rate and debt-to-equity ratio, directly impacting an enterprise’s cash flow health and financial stability. By improving asset liquidity and reducing debt levels, enterprises can reduce financial risks and enhance resilience against market shocks. Lastly, despite the relatively lower importance scores in the current model, factors such as net asset growth rate, total asset turnover ratio, annual incentive plans, and the proportion of management personnel should still be comprehensively considered. These indicators can also significantly impact an enterprise’s operations in certain circumstances. By establishing a comprehensive indicator monitoring system, enterprises can detect risk signals in time, take preventive measures, effectively control and reduce operational risks, and thus ensure the steady and sustainable development of the enterprise.

While this study has achieved notable accomplishments in enhancing the performance of corporate risk early warning systems, it acknowledges that the model has certain limitations in terms of generalization and adaptability. Currently, model construction and validation primarily rely on historical data, focusing mainly on specific financial indicators. To enhance the model’s adaptability to future market dynamics and different industries, future research can expand in the following directions. First, it is necessary to construct a more comprehensive framework for assessing corporate operational risks, incorporating non-financial indicators such as corporate culture and brand value, to more fully reflect the overall risk status of an enterprise. Second, the model’s dynamic adaptability and real-time warning capabilities should be improved by utilizing diverse data sources and real-time data. Lastly, it should explore the model’s applicability and adjustment strategies across different industries and enterprise sizes to ensure the model can effectively function over a broader range. Through these efforts, it is hoped that the corporate risk early warning system’s generalization and adaptability can be further enhanced, providing a more solid safeguard for the stable and sustainable development of enterprises.

## 5. Conclusion

This study extensively explores corporate operational risk warning strategies by constructing an improved RF model based on FCM clustering. The results show that the model is better than the traditional RF model in accuracy and response speed of risk early warning. Combining the risk indicator weight determined by the CRITIC weighting method with the preprocessing strategy of FCM clustering data, the model’s prediction ability and stability are effectively improved. The study identifies revenue growth rate and cash ratio as the two most critical indicators for assessing corporate operational risk, highlighting profitability, liquidity, innovation capability, and financial structure as indispensable factors in risk management. Despite significant achievements in enhancing the performance of the risk warning system, the study acknowledges several limitations. Firstly, the model construction and validation primarily rely on historical data, requiring further verification of its adaptability and generalization ability to future market dynamics. Secondly, the study focuses mainly on financial indicators, while the role of non-financial indicators such as corporate culture and brand value in risk assessment remains underexplored. This study pioneers the combination of the FCM clustering algorithm with the RF model, proposing a novel corporate operational risk early warning system. This innovative approach provides new perspectives and tools for research in this field. The FCM-RF model is less sensitive to noise and outliers, maintaining the stability of classification results and enhancing the model’s robustness compared to traditional RF models. The model proposed in this study can be widely applied in corporate risk management practices, assisting enterprises in timely identifying and addressing potential risks in complex market environments. By monitoring corporate financial indicators and promptly discovering financial risks, measures can be taken to reduce financial costs and improve the efficiency of capital usage. Through the FCM-RF model, enterprises can more accurately predict financial risks, especially cash flow risks. Enterprises can use the model’s predictive outcomes to adjust their asset structure in advance, increasing the proportion of liquid assets and reducing financial risks. When the model forecasts a period of tight cash flow, enterprises can sell part of their non-core assets in advance to ensure sufficient cash flow to cope with emergencies.

Although this study primarily focuses on financial indicators, the assessment of corporate operational risks should not be limited to these. The role of non-financial indicators such as corporate culture and brand value in risk assessment still warrants in-depth exploration. Future research can further investigate the impact of non-financial indicators, such as corporate culture, brand value, and employee satisfaction, on corporate operational risks. These indicators can offer a more comprehensive risk assessment perspective, helping enterprises better understand potential risk factors. With the continuous advancement of artificial intelligence and machine learning technologies, future research can integrate more advanced algorithms to enhance the intelligence level and predictive accuracy of risk early warning systems.

## Supporting information

S1 Data The relevant code in the manuscript can be found in the supporting information data file. (ZIP)
