# Supplementary material for: Early warning strategies for corporate operational risk: A study by an improved random forest algorithm using FCM clustering
Source: PLoS One. 2025 Mar 11;20(3):e0318491. doi: 10.1371/journal.pone.0318491 (PMC11896059; doi:10.1371/journal.pone.0318491)
Supplement: S1 Data — (ZIP) [file pone.0318491.s001.zip › 数据包/code description.docx]

**Code Explanation:** The code begins by using Scikit-learn's make_classification function to generate sample risk indicator data, which includes indicators such as market risk, R&D risk, financial risk, and human resource risk. Next, the CRITIC method is used to calculate the weights of these risk indicators. The CRITIC method involves standardizing the data, computing the standard deviation, correlation matrix, and information amount, and then calculating the CRITIC weights. Subsequently, the code employs fuzzy C-means (FCM) clustering to preprocess the data, assigning cluster labels to each data point. The purpose of clustering is to better group the data for subsequent modeling and analysis. The dataset is then split into a training set and a test set, with the training set used for model training and parameter tuning, and the test set used for model evaluation. For model parameter tuning, the code utilizes Grid Search to optimize the parameters of the Random Forest classifier. Grid Search searches for the best parameter combination within a given range and uses cross-validation to assess model performance, thereby obtaining the optimal model parameters. Finally, the best Random Forest classifier model obtained from Grid Search is used to evaluate the test set and generate a classification report, which includes metrics such as precision, recall, and F1-score for each category.

Modules to load: pip install numpy scipy matplotlib scikit-learn fuzzy-c-means

Development Environment: PyCharm Community Edition 2024.1

Programming Language: Python 3.8
